# Efficacy, safety, and lack of interactions with the use of raltegravir in HIV-infected patients undergoing antineoplastic chemotherapy

**DOI:** 10.7448/IAS.17.4.19590

**Published:** 2014-11-02

**Authors:** Sara Bañón, Isabel Machuca, Susana Araujo, Ana Moreno, María J Perez-Elías, Santiago Moreno, Jose Luis Casado

**Affiliations:** 1 Infectious Diseases, Ramon y Cajal Hospital, Madrid, Spain

## Abstract

**Introduction:**

Concomitant use of combination antiretroviral regimen (cART) and cancer chemotherapy is difficult due to complex interactions and increased toxicity. Raltegravir could be an adequate option through its favourable drug-drug interaction profile.

**Methods:**

Prospective longitudinal study of HIV patients with cancer, AIDS related or not, undergoing chemotherapy. Patients without resistance or previous failure were switched or initiated raltegravir plus two nucleoside analogues. Plasma trough levels of raltegravir were measured.

**Results:**

Overall, 28 patients receiving a raltegravir-based regimen (4 naive) with tenofovir-emtricitabine (18 cases) or abacavir-lamivudine (10 cases) were included. Mean age was 46.2 years (IQR, 39–52.7), and 79% were male. Median time of HIV was 201.7 months, CD4+ nadir was 268 cells/mm^3^, and 75% had previous AIDS. At the diagnosis of neoplasia, 17 were on protease inhibitors and 4 with efavirenz. Ten patients had a non-HIV-related cancer (three breast, two pancreatic, one Ewing sarcoma, one myeloblastic leukemia, one melanoma, one parotid adenocarcinoma, one lung), and 18 had an HIV-related cancer (nine non-Hodgkin lymphoma, seven Hodgkin disease, two anal). Overall, 43% of patients received more than one line of chemotherapy, including antimetabolites in 12 patients (5-FU, capecitabine, methotrexate, gemcitabine), alkylating agents in 12 cases (ciclophosphamide, iphosphamide), vinca alkaloids in 20 patients (vincristine, vinblastine, vindesine), antitumor antibiotics in 16 cases (adriamycin), cisplatin o carboplatin in six and monoclonal antibodies in six patients (rituximab, trastuzumab, cetuximab). Six patients modified the doses of antineoplastic agents due to toxicity (four neutropenia), not related to raltegravir. During a median follow up of 12.7 patients-year in concomitant therapy, there was only 1 case of virological failure and no patient discontinued raltegravir. Plasma concentrations of raltegravir in eight patients showed a median concentration of 143 ng/mL (79–455). Four patients (14%) died during the study, not related to AIDS progression. Raltegravir was continued after chemotherapy in all the cases.

**Conclusions:**

A raltegravir-based therapy is safe and effective in HIV patients undergoing antineoplastic chemotherapy, regardless of the type of tumour, and type and duration of chemotherapy. Pharmacokinetic data show adequate raltegravir levels.

